# Measuring symptoms severity in carpal tunnel syndrome: score agreement and responsiveness of the Atroshi-Lyrén 6-item symptoms scale and the Boston symptom severity scale

**DOI:** 10.1007/s11136-021-03039-1

**Published:** 2021-11-20

**Authors:** Kamelia Möllestam, Roberto S. Rosales, Per-Erik Lyrén, Isam Atroshi

**Affiliations:** 1 Department of Clinical Sciences Lund - Orthopedics, Lund University, Lund, Sweden; 2 Department of Orthopedics, Hässleholm-Kristianstad Hospitals, Hässleholm, 28125 Sweden; 3 Hand Surgery and Microsurgery Unit, GECOT, La Laguna, Tenerife, Spain; 4 Department of Applied Educational Science, Umeå University, Umeå, Sweden

**Keywords:** Carpal tunnel syndrome, Carpal tunnel release surgery, Item response theory, Patient-reported outcome measures, Symptom severity scale

## Abstract

**Purpose:**

To assess score agreement between the Atroshi-Lyrén 6-item symptoms scale and the Boston 11-item symptom severity scale and compare their responsiveness in patients with carpal tunnel syndrome before and after carpal tunnel release surgery.

**Methods:**

This prospective cohort study included 3 cohorts that completed the A-L and Boston scales (conventional score 1–5) on the same occasion: a preoperative and short-term postoperative cohort (212 patients), a mid-term postoperative cohort (101 patients), and a long-term postoperative cohort (124 patients). Agreement was assessed with Lin’s concordance correlation coefficient and Passing-Bablok regression analysis. Analyses using item response theory were conducted on responses from the preoperative/short-term postoperative cohort including testing of item infit/outfit. Reliability was assessed with Cronbach alpha. Overall and sex-specific effect sizes were calculated using Cohen’s d.

**Results:**

Lin’s CCCs were high (0.81–0.91). Passing-Bablok analysis showed constant and proportional differences in all cohorts except preoperative to short-term postoperative change. Both scales showed high reliability (alpha, 0.88–0.93). The IRT-based analyses showed infit/outfit values within the desired range. With IRT-based scoring, the A-L scale had significantly higher responsiveness than the Boston scale, overall (d, 2.02 vs 1.59), in women (d, 2.22 vs 1.77) and in men (d, 1.74 vs 1.36).

**Conclusion:**

The Atroshi-Lyrén 6-item symptoms scale and the Boston 11-item symptom severity scale show good agreement but are not equivalent in measuring CTS-related symptoms severity. When using IRT-based scoring, the Atroshi-Lyrén scale demonstrated significantly higher responsiveness.

**Supplementary Information:**

The online version contains supplementary material available at 10.1007/s11136-021-03039-1.

## Introduction

In patients with carpal tunnel syndrome (CTS), change in symptom severity is usually the most important treatment outcome and often used as primary endpoint in randomized clinical trials of treatment effectiveness [1–4]. Symptom severity is usually measured with patient-reported outcome measures. The Boston 11-item symptom severity scale, developed almost three decades ago, has been the most commonly used measure of symptom severity in CTS and has been translated to several languages [5–9]. In our previous research we investigated the Boston scale using modern measurement methodology based on item response theory (IRT) in a stepwise process that resulted in removal of 4 items that did not fit well in the scale and merging of 2 other items in that scale [10]. We developed a 6-item symptoms scale that demonstrated good internal consistency, test–retest reliability and validity in a comparison with the Boston scale and it did not exhibit differential item functioning with regard to gender [10]. The responsiveness of the 6-item scale has also been established [11]. Since its introduction, the Atroshi-Lyrén (A-L) 6-item CTS symptoms scale has been translated to various languages and used in clinical studies [12–14]. It is not known whether the scores of the Boston and the A-L scales are equivalent to enable direct score comparisons across studies that have used either scale. In addition, the two scales have not been compared with regard to responsiveness, which is probably the most clinically relevant psychometric property. When choosing a condition-specific patient-reported outcome measure for use in clinical research or practice it would be important to consider the responsiveness as well as efficiency of the measure. The purpose of this study was to assess the score agreement between the A-L 6-item CTS symptoms scale and the Boston 11-item symptom severity scale in patients with CTS before and after carpal tunnel release (CTR) surgery and compare their responsiveness. Since the A-L scale was developed using IRT, we hypothesized that the A-L scale would demonstrate higher responsiveness than the Boston scale.

## Materials and methods

We conducted a prospective cohort study at one orthopedic department in Southern Sweden. The department is the only facility in which carpal tunnel release surgery is performed in a region with 300,000 inhabitants. The study included data from 3 cohorts of patients who completed both scales on the same occasion. All patients were diagnosed with CTS by orthopedic or hand surgery specialists based on history and physical examination with or without nerve conduction tests. The inclusion criteria were a diagnosis of CTS and subsequent surgery with unilateral carpal tunnel release as the only procedure (ie, no concomitant procedures). All surgeries were done under local anesthesia and tourniquet. Postoperatively, a soft dressing was applied until suture removal at 12–14 days and patients received instructions about range of motion exercises and use of the hand as tolerated; hand therapy was not routinely prescribed.

### Study cohorts

**Fig. 1:**
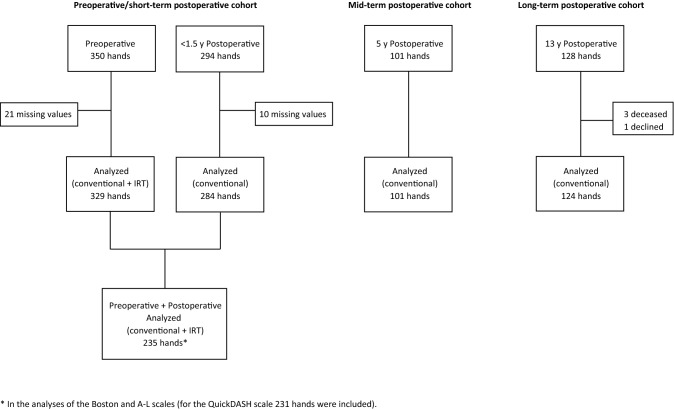
Flow chart of number of hands analyzed in the 3 cohorts

*Cohort 1 (preoperative/short-term postoperative cohort)* comprised 317 patients (329 hands) who completed the scales at the hospital either immediately before surgery or within 8 weeks before surgery, and 239 patients (284 hands) who completed the scales at 3 weeks to 17 months after surgery, from May 2017 through October 2018) (Fig. 1). Of these, 212 patients (235 hands) completed the scales both before and after surgery (Table 1). Postoperatively the scales were sent to the patients by mail. In patients who had surgery on both hands during the study period, the second surgery was usually performed after a minimum interval of 3 months.

**Table 1:** Characteristics of the patients in the 3 cohorts of patients with carpal tunnel syndrome treated with carpal tunnel release

Characteristic	Preoperative^a^	Short-term postoperative^b^	Mid-term postoperative	Long-term postoperative
Number of hands (patients)	329 (317)	284 (239)	101 (101)	124 (124)
Female sex, *n* (%)	202 (61)	174 (61)	76 (75)	93 (75)
Age at surgery, mean (SD) y	56 (15)	59 (15)	53 (11.5)	45 (8.6)
Dominant hand operated, *n* (%)	No data	No data	81 (80)	99 (80)
Right hand operated, *n* (%)	187 (57)	162 (57)	79 (78)	105 (85)
Time from surgery, mo				
Mean (SD)	NA	9.6 (4.4)	68 (8)	153 (15)
Median (range)	NA	7.4 (0.6–17)	69 (46–84)	149 (136–188)

^a^Scales completed immediately before surgery in 201 hands and within 8 weeks before surgery in 128 hands

^b^For 235 hands (212 patients) the scales were completed both preoperatively and postoperatively

*NA* Not applicable

*Cohort 2 (mid-term postoperative cohort)* comprised 101 patients (101 hands) who participated in a randomized placebo-controlled trial of steroid injection and subsequently had surgery [1]. The patients completed the scales as part of a follow-up conducted on all trial participants at 5 years after randomization [15]. The questionnaires were mailed to the patients. All trial participants provided follow-up data.

*Cohort 3 (long-term postoperative cohort)* comprised 124 patients who participated in a randomized controlled trial of open versus endoscopic CTR [16]. The patients completed both scales (questionnaires mailed to the patients) 12 to 14 years after surgery. Of the initial 128 participants 3 had died and 1 declined to answer the follow-up questionnaire.

In all 3 cohorts the questionnaires included the Boston scale, the A-L scale, and the 11-item disabilities of the arm, shoulder and hand (QuickDASH) scale. In the preoperative/short-term postoperative cohort, the Boston items in the preoperative questionnaire were placed first followed by the QuickDASH items and then the A-L items. In the postoperative questionnaire this order was used in 140 hands, but the two scales were placed in reverse order (separated by the QuickDASH) in 144 hands; this was randomly done according to surgery date. For the mid-term and long-term postoperative cohorts, the Boston items were placed before the A-L items separated by the QuickDASH.

### Scales

The Boston scale consists of 11 items that, referring to a specific side (right or left hand), inquire about the severity and frequency of pain, numbness and tingling as well as about other issues such as weakness and ability to hold small objects. Each item has 5 response choices score from 1 (no symptom) to 5 (most severe). The mean scale score is the mean value of all item scores. In conventional scoring a missing item response is usually replaced by the score mean for the other items. Although no rules exist about how many missing values are allowed for a conventional score to be computed, we allowed a maximum of 2 missing responses in this study. The final mean Boston symptom severity score may be any value between 1.0 (best) to 5.0 (worst).

The A-L scale consists of 6 items that inquire about severity and frequency of night and daytime numbness and tingling and pain (also referring to a specific side). Although the scale has been scored with IRT-based scoring it can also be scored conventionally on a scale from 1 (no symptoms) to 5 (most severe symptoms), with only 1 missing item response allowed with conventional scoring. Similarly, with conventional scoring the final mean A-L symptoms score may be any value between 1.0 (best) to 5.0 (worst).

The QuickDASH is an 11-item measure of upper extremity-related disability [17, 18]. The QuickDASH is not disease-specific but region-specific, and most items inquire about activity limitation. The QuickDASH score may range from 0 (no disability) to 100 (most severe disability), with only 1 missing item response allowed in conventional scoring.

### Missing data

The preoperative/short-term postoperative cohort initially comprised 350 hands preoperatively and 294 hands postoperatively, of which 21 and 10 respectively had missing values precluding calculation of a conventional score for one or both CTS scales and these were therefore excluded. No missing values occurred in the mid-term and long-term postoperative cohorts. Hands for which conventional CTS scores could be calculated but had 2 or more missing item responses on the QuickDASH were not excluded.

### Statistical analyses

The data from each of the 3 cohorts were analyzed separately (cohorts were not combined).

*Conventional scoring*: In each cohort we calculated the mean score and SD for all scales. We calculated the unadjusted difference between the Boston and A-L scale scores. In the preoperative/short-term postoperative cohort we also conducted a multivariate multiple linear regression analysis to determine whether age, sex, and time from surgery could be confounders in the difference of the change (preoperative to postoperative) scores between the Boston and A-L scales. We compared the scores according to the order in which the scales were placed in the questionnaire but found no differences and therefore we did not consider this factor further.

*Score agreement*: Absolute agreement in conventional scores between the Boston and A-L scales was assessed with Lin’s concordance correlation coefficient [19]. For assessing the difference in systematic error in measurement between the two scales, the Passing and Bablok regression analysis was used [20, 21].

*IRT analyses*: The IRT analyses were performed only on responses from the preoperative/short-term postoperative cohort. In the analyses of the Boston and A-L scales we used data from all hands with preoperative responses (*n* = 329), as well as data from the hands that had both preoperative and short-term postoperative responses (*n* = 235). In the analyses of the QuickDASH scale we included all hands with responses to at least 6 of the 11 items (*n* = 231).

In the development of the A-L scale [10] we used the partial credit model (PCM) as the item response model. However, based on the results of the development study we would expect the PCM to have a rather poor fit with the Boston scale items, and therefore, given the purpose of this study, we chose a more general model, the generalized partial credit model (GPCM) [22]. The PCM and GPCM are commonly used item response models for ordered polytomous data (ie, items that have multiple response options representing for example different levels of symptom severity). They model the probability of choosing the higher of two adjacent response options, conditional on the latent trait (symptom severity in this study). The GPCM also has a discrimination parameter that can be considered as an item weight; a larger discrimination parameter for a certain item implies a stronger association between the latent trait and the expected score on that item (ie, an item with a large discrimination parameter is better at distinguishing between lower and higher levels of symptom severity) (Appendix). We used the software ConQuest (version 4.0) for parameter estimation [23]. For parameter estimates in the GPCM we used weighted likelihood estimation (WLE) [24]. Parameter estimation was done in two steps. First, item parameters were estimated from the full preoperative data to maximize the amount of data in the item parameter estimation. In the second step, these pre-estimated item parameters were used to estimate preoperative and postoperative symptom severity for the hands that had both preoperative and postoperative responses. By following this procedure, we ensure that the item parameters are as robust as possible and that preoperative and postoperative symptom severity estimates are on the same scale. Item fit was evaluated using the infit and outfit statistics (Appendix). Expected values for both infit and outfit is 1, and it is desired that fit values should be approximately between 0.75 and 1.33, in accordance with Wilson [25].

*Reliability*: To analyze reliability we estimated the Cronbach alpha coefficient.

*Responsiveness*: We calculated the overall and sex-specific effect size for each scale in patients who had both preoperative and postoperative scores. As a measure of effects size we used Cohen’s d (mean difference in preoperative and postoperative scores divided by the pooled SD) with 95% CI. This was calculated for both the conventional and the IRT-based scores, using STATA version 16.0 (Stata Corporation, College Station, TX). We then compared d for the Boston and A-L scales using the z-test.

## Results

### Conventional scoring

In all 3 cohorts the mean A-L score was marginally (0.1 to 0.2 score units) lower than the mean Boston score (Table 2). The percentage mean difference (Boston as reference) was −9.8% at the short-term, −5.1% at the mid-term and −6.1% at the long-term follow-up. The multivariate multiple linear regression analysis of change scores among patients with both preoperative and short-term postoperative scores showed that age, sex and time since surgery were not confounders in the difference of the change (preoperative to postoperative) scores between the Boston and A-L scales.

**Table 2:** Scale scores (conventional scoring)

Scale	Preoperative mean (SD)	Short-term postoperative mean (SD)	Mid-term postoperative mean (SD)	Long-term postoperative mean (SD)
Boston	3.12 (0.76)	1.69 (0.75)	1.56 (0.66)	1.41 (0.65)
Atroshi-Lyrén	3.06 (0.84)	1.51 (0.72)	1.47 (0.66)	1.30 (0.55)
Score difference	−0.06 (0.40)	−0.19 (0.36)	−0.09 (0.28)	−0.11 (0.23)
QuickDASH	46 (22)	19 (20)	17 (20)	10 (16)

See Table 1 for number of hands in each cohort

Boston and Atroshi-Lyrén scale scores range from 1 (no symptoms) to 5 (most severe symptoms) and QuickDASH scale score ranges from 0 (no disability) to 100 (most severe disability)

### Score agreement

Lin’s concordance correlation coefficients were high, ranging from 0.81 to 0.91 (Table 3). The Passing-Bablok regression analysis showed a statistically significant constant and proportional difference in all cohorts, except in Cohort 1 preoperative-to-postoperative change where it showed constant agreement but proportional disagreement.

**Table 3:** Concordance correlation and Passing-Bablok agreement between the Boston and Atroshi-Lyrén scales

	Preoperative	Short-term postoperative	Short-term change	Mid-term postoperative	Long-term postoperative
CCC	0.87 (0.85 to 0.89)	0.85 (0.82 to 0.89)	0.81 (0.77 to 0.86)	0.90 (0.87 to 0.94)	0.91 (0.89 to 0.94)
A	−0.33 (−0.58 to −0.19)	0.21 (0.06 to 0.29)	0.07 (−0.03 to 0.18)	0.08 (0.01 to 0.25)	0.31 (0.23 to 0.39)
B	1.10 (1.05 to 1.17)	0.77 (0.71 to 0.86)	1.10 (1.01 to 1.17)	0.92 (0.75 to 0.99)	0.69 (0.61 to 0.77)

Values within parentheses are 95% confidence intervals (CI)

A is the constant difference and B the proportional difference between the two scales, a CI including 0 or 1 implies constant or proportional agreement, respectively

*CCC* Lin’s concordance correlation coefficient

### IRT

All item infit and outfit values for the Boston and A-L scales were within the 0.75–1.33 range, indicating acceptable fit (Table 4). All QuickDASH items showed acceptable fit, with values ranging from 0.98 to 1.05. The mean Boston score was −0.12 (SD 1.02) preoperatively and −1.68 (SD 0.94) postoperatively, and the mean A-L score was −0.03 (SD 1.02) preoperatively and −2.33 (SD 1.24) postoperatively.

**Table 4:** Infit and outfit values for average item locations in the Boston and Atroshi-Lyrén (A-L) scales in the item response theory (IRT)-based analyses

		Boston				A-L	
		Preoperative				Preoperative	
Item		Infit	Outfit	Item		Infit	Outfit
1	Pain—night, severity	1.02	1.01	1	Pain—night, severity	0.97	0.94
2	Pain—wakening, frequency	1.00	0.97	2	Pain—daytime, severity	1.00	0.99
3	Pain—daytime, severity	1.02	1.01	3	Numbness/tingling—night, severity	0.99	0.96
4	Pain—daytime, frequency	1.01	1.01	4	Numbness/tingling—daytime, severity	0.99	0.99
5	Pain—daytime, duration	1.03	1.10	5	Pain—wakening, frequency	1.02	1.02
6	Numbness—severity	1.03	1.04	6	Numbness/tingling—wakening, frequency	1.00	0.98
7	Weakness—severity	1.01	1.01				
8	Tingling—severity	1.01	1.01				
9	Numbness/tingling—night, severity	1.03	1.01				
10	Numbness/tingling—wakening, frequency	1.02	1.00				
11	Gripping small objects	0.99	1.02				

### Reliability

The Cronbach alpha estimates were high for all scales. The preoperative and postoperative alpha for the Boston scale were 0.88 and 0.93, for the Atroshi-Lyrén scale 0.89 and 0.91 and for the QuickDASH 0.91 and 0.94.

### Responsiveness

Cohen’s d was large for all scales (> 1.3 for the CTS scales and > 0.9 for the QuickDASH) with both conventional and IRT-based scoring (Table 5). The A-L scale had larger overall and sex-specific d values than the Boston scale with both conventional and IRT-based scoring; the A-L scale’s larger IRT-based d values were statistically significant (*p* < 0.001). For all scales d was smaller among men than among women.

**Table 5:** Overall and sex-specific responsiveness of the scales

	Conventional scoring			IRT scoring		
Scale	Preoperative mean (SD)	Postoperative mean (SD)	d (95% CI)	Preoperative mean (SD)	Postoperative mean (SD)	d (95% CI)
All hands (*n* = 235)						
Boston	3.03 (0.72)	1.68 (0.73)	1.86 (1.64–2.08)	−0.12 (1.02)	−1.68 (0.94)	1.59 (1.38–1.80)
A-L	2.98 (0.82)	1.47 (0.68)	2.00 (1.77–2.21)	−0.03 (1.02)	−2.33 (1.24)	2.02 (1.79–2.24)^d^
QuickDASH^a^	44 (22)	18 (19)	1.25 (1.04–1.45)	−0.15 (1.24)	−1.82 (1.55)	1.19 (1.00–1.39)
Women (*n* = 141)^b^						
Boston	3.05 (0.71)	1.59 (0.65)	2.14 (1.84–2.43)	−0.10 (1.01)	−1.76 (0.86)	1.77 (1.49–2.04)
A-L	3.01 (0.81)	1.40 (0.62)	2.22 (1.93–2.52)	−0.01 (1.03)	−2.46 (1.17)	2.22 (1.92–2.52)^d^
QuickDASH^a^	47 (22)	17 (19)	1.47 (1.19–1.74)	0.05 (1.21)	−1.78 (1.49)	1.35 (1.09–1.61)
Men (*n* = 94)^c^						
Boston	3.0 (0.74)	1.82 (0.81)	1.52 (1.20–1.85)	−0.15 (1.04)	−1.55 (1.03)	1.36 (1.04–1.67)
A-L	2.94 (0.84)	1.58 (0.76)	1.70 (1.37–2.04)	−0.06 (1.01)	−2.12 (1.33)	1.74 (1.41–2.08)^d^
QuickDASH^a^	39 (21)	19 (20)	0.95 (0.65–1.26)	−0.44 (1.24)	−1.89 (1.64)	0.99 (0.69–1.30)

Boston and Atroshi-Lyrén (A-L) scale scores range from 1 (no symptoms) to 5 (most severe) and the QuickDASH from 0 (no disability) to 100 (most severe)

Cohen’s d values shown as absolute values

^a^No conventional QuickDASH scores because of ≥ 2 missing items in 11 women hands (5 preoperative and 6 postoperative) and 3 men hands (2 preoperative and 1 postoperative); 4 hands (3 women and 1 man) with ≥ 5 missing QuickDASH items were not included in the IRT analyses

^b^Right hand operated on in 82 (58%), mean time (weeks) from surgery 30.7 (SD 15.5)

^c^Right hand operated on in 54 (57%), mean time (weeks) from surgery 30.6 (SD 15.2)

^d^*p* < 0.001 (A-L vs Boston)

## Discussion

Our study shows that in patients with CTS the mean conventional scores for the A-L scale were consistently lower than the Boston scale by 0.1 to 0.2 score units before and after surgery. However, this small difference is not considered clinically relevant. Thus, in pre-post intervention study design the mean change scores for both scales are expected to be near to equivalent. In a previous study, high agreement was found between these scales with an intraclass correlation coefficient of 0.80 [10]. Agreement analysis in this study demonstrated that, despite a high absolute agreement by Lin’s CCC (0.81–0.91), the Passing-Bablok analyses showed constant and proportional differences in almost all comparisons. However, there was constant agreement between the two scales in the change in scores from baseline to short-term postoperatively, and the proportional difference was only marginally statistically significant.

The A-L scale has previously demonstrated good construct validity and reliability for measuring symptoms severity in CTS, both after surgical treatment [10, 14] and after steroid injection [12]. This was also the case in our present study (Cronbach alpha estimates 0.89–0.91). A study that used IRT (Rasch model) to compare the A-L and Boston scales in patients with surgically treated CTS suggested that the A-L scale had better psychometric properties based on analysis of item thresholds and differential item functioning [14].

In the present study all the Boston items had good infit when estimated from the preoperative data, which was not the case when the A-L scale was developed. However, this is not surprising given that we use a more general model in this study. In that development work, the starting point was the Boston scale items estimated on preoperative data. However, in that work two items were removed from the Boston scale before item parameter estimation (based on factor analysis), and that might have altered the scale enough to explain the difference in fit patterns. Another surprising finding was the coefficient alpha estimates being higher for the postoperative data than the preoperative data for all scales. This is counterintuitive as the spread (SD) is smaller in the postoperative measures. However, the results may be due to the presence of outliers, as it has been found that coefficient alpha estimates can be severely inflated by outliers in rating scale item responses [26].

If the two scales are not equivalent, which one should be used for measuring symptoms severity related to CTS? A scale may be highly reliable and valid but with low responsiveness [21]. We believe that it would be advantageous to use the scale that has demonstrated higher responsiveness. Our study showed that the A-L scale had higher responsiveness than the Boston scale especially when using IRT-based scoring. The fact that many Boston items did not fit the PCM in the original study [10] may be indicative of “noise”, which could be a plausible explanation to its lower responsiveness. Another advantage of the IRT-based scoring is that it can use data even from patients with more missing responses than allowed with conventional scoring. Considering that responsiveness of the A-L was high with both scoring methods, conventional scoring would be more feasible in clinical practice.

There was a statistically significant difference in both overall and sex-specific responsiveness between the A-L and Boston scales when using IRT-based scoring. Previous studies of both scales have shown no differential item functioning with regard to sex [10, 27]. In this study the QuickDASH had similar responsiveness with both conventional and IRT-based scoring. It is important to note that even though the QuickDASH had good item fit and reliability, that is, the scale meets some of the criteria to measure an intended construct, it is not specifically intended to measure CTS symptoms severity. A previous study has also shown higher responsiveness of the A-L scale than the QuickDASH, probably because the former is a disease-specific measure of symptoms [11].

One limitation is that our study only involved patients whose symptoms severity required surgical treatment. Thus, our results are generalizable mainly to surgically treated patients.

In conclusion, our study shows good agreement between the A-L 6-item CTS symptoms scale and the Boston 11-item symptom severity scale in measuring symptoms severity related to CTS. When using IRT-based scoring, the A-L scale demonstrated significantly higher responsiveness than the Boston scale. As the more responsive and efficient measure, the A-L scale would be the preferable measure when evaluating symptoms severity in CTS.

## Supplementary Information

Below is the link to the electronic supplementary material.

Supplementary file1 (PDF 410 kb)

## Data Availability

The datasets generated and/or analyzed during the current study are not publicly available due to that individual patient privacy could be compromised but are available from the corresponding author on reasonable request.

## References

[1] Atroshi I, Flondell M, Hofer M, Ranstam J (2013). Methylprednisolone injections for the carpal tunnel syndrome: A randomized, placebo-controlled trial. Annals of internal medicine.

[2] Atroshi I, Larsson GU, Ornstein E, Hofer M, Johnsson R, Ranstam J (2006). Outcomes of endoscopic surgery compared with open surgery for carpal tunnel syndrome among employed patients: Randomised controlled trial. BMJ (Clinical research ed.).

[3] Gerritsen AA, de Vet HC, Scholten RJ, Bertelsmann FW, de Krom MC, Bouter LM (2002). Splinting vs surgery in the treatment of carpal tunnel syndrome: A randomized controlled trial. JAMA.

[4] Jarvik JG, Comstock BA, Kliot M, Turner JA, Chan L, Heagerty PJ, Hollingworth W, Kerrigan CL, Deyo RA (2009). Surgery versus non-surgical therapy for carpal tunnel syndrome: A randomised parallel-group trial. Lancet (London, England).

[5] Atroshi I, Johnsson R, Sprinchorn A (1998). Self-administered outcome instrument in carpal tunnel syndrome. Reliability, validity and responsiveness evaluated in 102 patients. Acta orthopaedica Scandinavica.

[6] Leite JC, Jerosch-Herold C, Song F (2006). A systematic review of the psychometric properties of the Boston Carpal Tunnel Questionnaire. BMC Musculoskeletal Disorders.

[7] Levine DW, Simmons BP, Koris MJ, Daltroy LH, Hohl GG, Fossel AH, Katz JN (1993). A self-administered questionnaire for the assessment of severity of symptoms and functional status in carpal tunnel syndrome. The Journal of Bone and Joint Surgery.

[8] Mondelli M, Reale F, Sicurelli F, Padua L (2000). Relationship between the self-administered Boston questionnaire and electrophysiological findings in follow-up of surgically-treated carpal tunnel syndrome. Journal of hand surgery (Edinburgh, Scotland).

[9] Rosales RS, Delgado EB, Díez de la Lastra-Bosch I (2002). Evaluation of the Spanish version of the DASH and carpal tunnel syndrome health-related quality-of-life instruments: Cross-cultural adaptation process and reliability. The Journal of hand surgery.

[10] Atroshi I, Lyrén PE, Gummesson C (2009). The 6-item CTS symptoms scale: A brief outcomes measure for carpal tunnel syndrome. Quality of Life Research.

[11] Lyrén PE, Atroshi I (2012). Using item response theory improved responsiveness of patient-reported outcomes measures in carpal tunnel syndrome. Journal of Clinical Epidemiology.

[12] Craw JR, Church DJ, Hutchison RL (2015). Prospective comparison of the six-item carpal tunnel symptoms scale and portable nerve conduction testing in measuring the outcomes of treatment of carpal tunnel syndrome with steroid injection. Hand (New York, NY).

[13] Matsuo RP, Fernandes CH, Meirelles LM, Raduan Neto J, Dos Santos JB, Fallopa F (2016). Translation and cross-cultural adaptation of the 6-item carpal tunnel syndrome symptoms scale and palmar pain scale questionnaire Into Brazilian Portuguese. Hand (New York, NY).

[14] Multanen J, Ylinen J, Karjalainen T, Ikonen J, Häkkinen A, Repo JP (2020). Structural validity of the Boston Carpal Tunnel Questionnaire and its short version, the 6-Item CTS symptoms scale: A Rasch analysis one year after surgery. BMC Musculoskeletal Disorders.

[15] Hofer M, Ranstam J, Atroshi I (2021). Extended follow-up of local steroid injection for carpal tunnel syndrome: A randomized clinical trial. JAMA Network Open.

[16] Atroshi I, Hofer M, Larsson GU, Ranstam J (2015). Extended follow-up of a randomized clinical trial of open vs endoscopic release surgery for carpal tunnel syndrome. JAMA.

[17] Beaton DE, Wright JG, Katz JN, Upper Extremity Collaborative Group (2005). Development of the QuickDASH: Comparison of three item-reduction approaches. The Journal of Bone and Joint Surgery.

[18] Gummesson C, Ward MM, Atroshi I (2006). The shortened disabilities of the arm, shoulder and hand questionnaire (QuickDASH): Validity and reliability based on responses within the full-length DASH. BMC Musculoskeletal Disorders.

[19] Lin LI (1992). Assay validation using the concordance correlation coefficient. Biometrics.

[20] Passing H, Bablok W (1984). Comparison of several regression procedures for method comparison studies and determination of sample sizes. Application of linear regression procedures for method comparison studies in Clinical Chemistry, Part II. Journal of Clinical Chemistry and Clinical Biochemistry.

[21] Rosales RS, Atroshi I (2020). The methodological requirements for clinical examination and patient-reported outcomes, and how to test them. The Journal of Hand Surgery, European.

[22] Muraki E (1992). A generalized partial credit model: Application of an EM algorithm. Applied Psychological Measurement.

[23] Wu, M. L., Adams, R. J., & Wilson, M. (2007). ConQuest generalized item response modeling software. Australian Council for Educational Research (ACER).

[24] Park C, Muraki E, Yanai H, Okada A, Shigemasu K, Kano Y, Meulman JJ (2003). Bias of ability estimates using Warm’s weighted likelihood estimator (WLE) in the generalized partial credit model (GPCM). New developments in psychometrics.

[25] Wilson M (2005). Constructing measures. An item response modeling approach.

[26] Liu Y, Wu AD, Zumbo BD (2010). The impact of outliers on Cronbach’s coefficient alpha estimate of reliability: ordinal/rating scale item responses. Educational and Psychological Measurement.

[27] Jerosch-Herold C, Bland J, Horton M (2021). Is it time to revisit the Boston Carpal Tunnel Questionnaire? New insights from a Rasch model analysis. Muscle & Nerve.

