# Supplementary material for: Measuring symptoms severity in carpal tunnel syndrome: score agreement and responsiveness of the Atroshi-Lyrén 6-item symptoms scale and the Boston symptom severity scale
Source: Qual Life Res. 2021 Nov 20;31(5):1553–60. doi: 10.1007/s11136-021-03039-1 (PMC9023404; doi:10.1007/s11136-021-03039-1)
Supplement: Supplementary file 1 — Supplementary file1 (PDF 410 kb) [file 11136_2021_3039_MOESM1_ESM.pdf]

Measuring symptoms severity in carpal tunnel syndrome: score agreement and responsiveness of the Atroshi-Lyrén 6-item symptoms scale and the Boston symptom severity scale

Kamelia Möllestam, MD,[1,2] Roberto S. Rosales, MD, PhD,[3] Per-Erik Lyrén, PhD,[4] Isam Atroshi, MD, PhD[1,2]

[1] Department of Clinical Sciences Lund - Orthopedics, Lund University, Lund, Sweden.

[2] Department of Orthopedics, Hässleholm-Kristianstad Hospitals, Hässleholm, Sweden.

[3] Hand Surgery and Microsurgery Unit, GECOT, La Laguna, Tenerife, Spain.

[4] Department of Applied Educational Science, Umeå University, Umeå, Sweden.

Correspondent author:

Kamelia Möllestam, kamelia.mollestam@skane.se

Appendix. Explanation of the IRT methods and statistics.

IRT models

IRT models are statistical models of the relationship between a person's score on the latent trait being measured and the probability of choosing each response on each item measuring that trait. The partial credit model (PCM) and the generalized partial credit model (GPCM) are commonly used IRT models for ordered polytomous data (ie, items that have different numbers of response options). They both model the probability of choosing the higher of two adjacent response options, conditional on the latent trait (symptom severity in this study). Both models have a set of threshold parameters describing the item location (or difficulty), that is, how likely it is to report problems on the item. However, the GPCM also has a discrimination parameter that can be viewed as an item weight. A larger discrimination parameter for a certain item implies a stronger association between the latent trait and the expected score on that item. This is in contrast to the PCM, in which the discrimination parameter is fixed at 1 for all items.

Infit and outfit statistics

Infit and outfit are chi-square-like statistics (squared standardized residuals); the difference is that infit is based on information weighted residuals while outfit is not. This means that infit is sensitive to unexpected responses (according to the model) near the person's location on the scale (symptom severity in this case), while outfit is more sensitive to unexpected responses further away from the person's location on the scale (i.e. outlier responses). Consequently, infit values are of greater concern, as outfit values can be highly influenced by a few outliers.
